# The Impact of Endocrine Disruptor Exposure During Pregnancy on Bacterial Complications and Viral Infections: A Narrative Review

**DOI:** 10.3390/microorganisms14051012

**Published:** 2026-04-30

**Authors:** Sofoklis Stavros, Angeliki Gerede, Nektaria Zagorianakou, Efthalia Moustakli, Anastasios Potiris, Ismini Anagnostaki, Alexios Kozonis, Maria Tzeli, Aikaterini Lydia Vogiatzoglou, Pavlos Machairoudias, Konstantinos Zacharis, Athanasios Zikopoulos, Dimitrios Loutradis, Ekaterini Domali

**Affiliations:** 1 Third Department of Obstetrics and Gynecology, University General Hospital “ATTIKON”, Medical School, National and Kapodistrian University of Athens, 12462 Athens, Greece; apotiris@med.uoa.gr (A.P.); alkozonis@med.uoa.gr (A.K.); vogiatzoglou.lydia@gmail.com (A.L.V.); 2 Unit of Maternal-Fetal-Medicine, Department of Obstetrics and Gynecology, Medical School, Democritus University of Thrace, 68100 Alexandroupolis, Greece; agerede@otenet.gr; 3 Department of Nursing, School of Health Sciences, University of Ioannina, 4th Kilometer National Highway Str. Ioannina-Athens, 45500 Ioannina, Greece; zagorianakou@uoi.gr (N.Z.); ef.moustakli@uoi.gr (E.M.); 4 Scientific Laboratory for Innovative Technologies in Internal Medicine, Preventive Medicine and Overall Care, 45500 Ioannina, Greece; 5 Medical School, National and Kapodistrian University of Athens, 11528 Athens, Greece; isanagnostaki3@gmail.com; 6 Department of Midwifery, Faculty of Health and Caring Sciences, University of West Attica, 12243 Athens, Greece; mtzeli@uniwa.gr; 7 Oxford University Hospitals NHS Foundation Trust, Oxford OX3 9DU, UK; paul.machaioudias@ouh.nhs.uk; 8 Department of Obstetrics and Gynecology, General Hospital of Lamia, 35100 Lamia, Greece; zaxarisk@yahoo.com; 9 Department of Reproductive Medicine and Surgery, University College London Hospitals NHS Foundation Trust, 235 Euston Road, London NW1 2BU, UK; thanzik92@gmail.com; 10 Diagnostic and Therapeutic Fertility Institute S.A, 11521 Athens, Greece; loutradi@otenet.gr; 11 First Department of Obstetrics and Gynecology, Alexandra Hospital, Medical School, National and Kapodistrian University of Athens, 11528 Athens, Greece; kdomali@yahoo.fr

**Keywords:** maternal microbiota, immune modulation, dysbiosis, placental transfer, oxidative stress, host–microbe interactions, immunotoxicity, microbial diversity, inflammatory pathways

## Abstract

Endocrine-disrupting chemicals (EDCs) are a diverse group of environmental pollutants capable of interfering with hormonal and immune system regulation. In recent years, increasing concern has been raised about the effects of chemicals, including bisphenols, phthalates, per- and polyfluoroalkyl substances (PFAS), insecticides, and parabens, on maternal and fetal health, primarily due to their widespread exposure in human populations. Pregnancy represents a critical window characterized by tightly regulated hormonal and immunological adaptations. Emerging evidence suggests that EDC exposure during this period may alter maternal microbiota, disrupt immune responses, and interfere with endocrine signaling. These changes may increase susceptibility to bacterial and viral infections, including bacterial vaginosis, urinary tract infections, and intrauterine infections, all of which are associated with adverse pregnancy outcomes. This review summarizes the current evidence on the sources and mechanisms of exposure to endocrine disruptors during pregnancy and examines the potential biological pathways linking endocrine disruption to the development of infections. Particular emphasis is placed on the interactions between immune regulation, hormonal signaling, and changes in the microbiome, which may contribute to increased susceptibility to infections. A deeper understanding of these complex mechanisms is critical to improve risk assessment, develop effective public health strategies, and ultimately protect maternal and fetal health in an environment of increasing chemical exposure. A literature search was conducted using PubMed/MEDLINE, Scopus, and Web of Science, including studies published up to January 2026.

## 1. Introduction

Exposure to environmental chemicals that interfere with hormonal function represents a growing global public health concern [1]. The chemicals that affect hormones are called EDCs, as they disrupt the typical functions of the endocrine system by interfering with how hormones send and receive signals within the body [1,2,3]. EDCs are present in a wide variety of consumer products, including plastics, food packaging, cosmetics, pesticides, industrial chemicals, and various household products, and have a broad distribution throughout the environment. The majority of EDCs that have been studied in depth include common compounds such as bisphenol (BPA), phthalates, perfluoroalkyl and polyfluoroalkyl substances (PFAs), parabens, dioxins, and organochlorine pesticides (OCPs). Due to their widespread use and persistence in the environment, humans are continuously exposed to EDCs through diet, inhalation, and dermal contact [4,5,6].

Pregnancy represents a critical period of exposure, characterized by complex hormonal and immunological changes that are essential for both fetal development and maternal health [7]. For instance, hormones such as progesterone, estrogen, and human chorionic gonadotropin increase immunological tolerance to the fetus, control metabolism, and promote placental development. However, the presence of endocrine disruptors may disrupt these regulatory processes [8]. EDCs can bind to hormone receptors, altering gene expression and epigenetic processes and mimicking endogenous hormones. This may lead to preterm delivery, restriction of fetal development, metabolic problems, and developmental issues [9].

In addition to endocrine-disrupting activities, there are indications of the immunological effects of EDCs [10]. Pregnancy has been associated with a balance between tolerance to the fetus and immune competence against infections. This immune competence depends on immune cells, cytokines, and inflammatory responses. Interruption of these processes may influence the risk of infections in pregnant women [11,12]. Experimental studies demonstrate that EDCs modulate immune function by altering cytokine production, oxidative stress (OS) pathways, and immune cell activity, thereby potentially compromising host defense against infections [13].

Another important and emerging area of research is the link between EDCs and the maternal microbiome. The microbiome maintains physiological homeostasis, supports immune function, and protects against pathogenic microorganisms [14]. During pregnancy, significant alterations in the gut and vaginal microbiota occur, which are important for the normal course of pregnancy. Alterations in the microbiome have been linked to bacterial vaginosis, urinary tract infections, preterm labor, and intrauterine inflammation. Recent studies indicate that environmental pollutants can modulate microbial diversity and metabolism, thereby altering host–microbe interactions and increasing susceptibility to infection [15,16].

Infections associated with pregnancy, both bacterial and viral, remain a cause of morbidity among pregnant women. Bacterial infections include bacterial vaginosis, urinary tract infections, and infections caused by bacteria such as *Escherichia coli*, group B *Streptococcus*, and *Ureaplasma* species. These infections have the potential to cause complications like preterm labor, chorioamnionitis, and neonatal sepsis [17,18]. Other infections include cytomegalovirus, influenza virus, Zika virus, and SARS-CoV-2, all of which have the potential to cause complications among pregnant women. Thus, factors influencing immune defense or the balance of the microbial flora may have a role in the development of infections during pregnancy [17,19].

Despite growing interest in the effects of EDCs on the endocrine system, immune regulation, and the microbiome, the mechanisms underlying their association with increased susceptibility to infections during pregnancy remain unclear. These complex interactions require a holistic approach, integrating all the above fields of study.

The purpose of this review is to provide a comprehensive and integrative overview of the effects of endocrine-disrupting chemicals during pregnancy, with a particular focus on susceptibility to bacterial and viral infections. This work aims to integrate these domains into a single, mechanistic framework, in contrast to earlier reviews that primarily addressed endocrine, immunological, or microbiome-related effects independently. It highlights how endocrine disruption may lead to immune dysregulation and microbiome alterations, collectively increasing the risk of infection. In addition, this review identifies key gaps in current knowledge, particularly regarding the interaction between environmental exposures, host immune responses, and microbial dynamics during pregnancy. By synthesizing evidence across these interconnected systems, this work seeks to advance understanding of the complex biological pathways linking EDC exposure to infectious complications in pregnancy.

## 2. Literature Search Strategy

This narrative review was conducted through a comprehensive literature search aimed at identifying studies examining the effects of EDCs on immune function, microbiome alterations, and susceptibility to bacterial and viral infections during pregnancy.

A systematic search of the literature was performed using electronic databases, including PubMed/MEDLINE, Scopus, and Web of Science. The search included studies published up to January 2026. Initial searches using broad combinations of terms related to endocrine-disrupting chemicals (e.g., “endocrine-disrupting chemicals”, “bisphenol A”, “phthalates”, “PFAS”, “persistent organic pollutants”) and pregnancy (e.g., “pregnancy”, “maternal exposure”) yielded approximately 7000 records in PubMed and 8900 records in Scopus. Web of Science was additionally used for complementary and targeted searches.

Representative search strategies included combinations of keywords using Boolean operators, such as: (“endocrine-disrupting chemicals” OR “bisphenol A” OR “phthalates” OR “PFAS” OR “persistent organic pollutants”) AND (“pregnancy” OR “maternal exposure”) AND (“microbiome” OR “immune response” OR “infection”). Search parameters were adapted for each database.

Additional targeted searches were conducted to identify studies addressing specific mechanisms and outcomes, including “microbiome”, “immune response”, “bacterial infections”, and “viral infections” (e.g., cytomegalovirus, influenza, Zika virus, SARS-CoV-2).

Eligible studies included original research articles, epidemiological studies, experimental studies, and relevant review articles focusing on human populations, animal models, and mechanistic pathways. Particular emphasis was placed on studies investigating pregnancy-related outcomes, immune modulation, microbiome alterations, and infection susceptibility.

Studies were selected based on relevance to the topic, scientific quality, and contribution to understanding the relationship between EDC exposure and infection risk during pregnancy. Screening and selection were performed through evaluation of titles, abstracts, and full texts by the authors, followed by consensus discussion. No strict exclusion criteria were applied, consistent with the narrative nature of this review; however, priority was given to recent and high-quality studies. Additional articles were identified through manual screening of reference lists of selected publications.

A comprehensive review or meta-analysis was deemed impractical due to the heterogeneity of the available data, which included variations in research design, exposure assessment, outcome definitions, and model systems (human, animal, and in vitro). To synthesize findings across diverse study designs, a narrative review approach was adopted. A comprehensive search was conducted across multiple databases, and studies were ranked based on methodological quality, relevance, and recency to minimize selection bias.

In total, 160 references were incorporated into the final narrative synthesis, including original research articles, experimental studies, epidemiological investigations, and relevant review articles.

The collected evidence was synthesized to provide an integrated overview of the current knowledge on the interactions between endocrine disruption, immune regulation, microbiome changes, and infectious disease susceptibility during pregnancy.

## 3. EDCs: Sources and Exposure During Pregnancy

EDCs are a group of pollutants that are widespread in the environment and have the potential to impact the body’s regular functions through interference in the endocrine system. EDCs are present in a wide range of products, agricultural products, and the environment in general. Due to their widespread use and availability, human exposure is continuous and occurs through the intake of food products, respiration, and dermal contact [20,21]. Pregnancy is a time of intricate endocrine and metabolic changes that are necessary for the regular development of the fetus and the health of the mother. Therefore, the mother is more susceptible to the effects of these chemicals.

Recent studies have demonstrated that EDCs influence microbial balance, immune responses, and hormonal regulation in pregnant women. The ability of these chemicals to bioaccumulate in the body and cross the placenta makes the fetus susceptible to harmful factors [22,23]. Therefore, understanding the impact of endocrine disruptors on susceptibility to viral infections and bacterial complications during pregnancy requires a comprehensive understanding of their sources, mechanisms of action, and routes of exposure

### 3.1. Definition and Mechanisms of Endocrine Disruption

EDCs are external substances or mixtures that can disrupt the endocrine system and may cause harmful effects in organisms, their offspring, or populations. EDCs have the ability to mimic the function of hormones, inhibit the binding of hormones to their receptors, modify the synthesis and metabolism of hormones, and modify the excretion of hormones from the body [24,25].

Many endocrine disruptors affect the endocrine system by interacting with hormone receptors such as estrogen, androgen, progesterone, and thyroid receptors. These interactions may either stimulate or inhibit receptor function, depending on the specific compound. Bisphenol A is a substance that is known to stimulate the function of the estrogen receptor [26].

EDCs may also affect endocrine function through epigenetic mechanisms, including DNA methylation, histone modifications, and miRNA regulation. These epigenetic modifications may alter endocrine function, thereby leading to developmental and immunological effects [27]. Furthermore, certain endocrine-disrupting chemicals have been demonstrated to modulate inflammatory responses and OS, thereby further disrupting endocrine function and contributing to adverse pregnancy outcomes [25].

Endocrine disruption may also influence the immune system. Hormonal regulation plays a significant role in controlling immune function, and disturbances in hormone levels may impair immune responses. Immune function is critical during pregnancy, as a balance between tolerance and immunity must be maintained [28].

### 3.2. Major Classes of EDCs Relevant to Pregnancy

Many chemicals have been identified as endocrine disruptors, but some key categories are of particular concern due to their widespread use and persistence in the environment, making them significant sources of exposure for pregnant women and their fetuses [25,29].

Bisphenols, particularly BPA, are widely used in polycarbonate plastics and epoxy resins in food containers, water bottles, and heat seals. BPA has been identified in biological samples, including maternal blood, urine, amniotic fluid, and placental tissue, and has been shown to exhibit estrogenic activity that disrupts hormonal balance [30,31].

Another significant group is phthalates, which are used as plasticizers for plastics. Phthalates are used in food containers, medical equipment, cosmetics, and personal care products. Some of them are associated with anti-androgenic activity and are thought to influence both reproductive and immune system development [32,33].

Special emphasis has been placed on per- and polyfluoroalkyl substances (PFAS), which are used in non-stick cookware, stain-resistant clothing, and fire-fighting foams. PFAS are characterized by their persistence and long residence time in the body, leading to repeated exposure. They are associated with compromised immune response, decreased vaccine efficacy, and metabolic disorders [34].

Pesticides are another significant group of endocrine disruptors. These include chemicals such as organophosphates, carbamates, and organochlorine insecticides, which are frequently used in agricultural settings and may leach into the food supply, soil, and water resources. Pesticides have been shown to affect thyroid hormone function, as well as estrogenic and androgenic signaling [5,35].

Finally, persistent organic pollutants (POPs) such as PCBs and dioxins are byproducts of industrial processes and waste incineration. Due to their lipophilic properties, these chemicals are absorbed in adipose tissue and have a prolonged residence time in the body [36].

Overall, these endocrine disruptors contribute to environmental exposure and may affect the endocrine and immune systems in pregnant women, increasing the risk of infections. Table 1 summarizes major EDC classes, sources, and pregnancy-relevant effects.

### 3.3. Routes of Maternal Exposure

Due to their ubiquitous presence in the environment and consumer products, humans are exposed to EDCs through multiple pathways. Diet is one of the main routes of exposure to EDCs, as many EDCs are known to leach into food and beverages from packaging materials. In addition, diet includes seafood, water, and agricultural products that are contaminated with EDCs [21,46].

Another significant route of exposure to EDCs is through inhalation. Various pollutants present in dust, air, and work environments, including those where pesticides and chemicals are used, are inhaled into the body. Some chemicals evaporate and become trapped in indoor air [47,48].

Dermal absorption is another significant route, especially for chemicals used in cosmetics, cleaning agents, and personal care products. These include phthalates and parabens, which can be absorbed and enter the bloodstream [32,49].

Furthermore, occupational exposure constitutes a significant risk factor, particularly among pregnant women employed in industrial, agricultural, and healthcare settings with frequent chemical exposure. There is also the risk of chronic exposure at low levels in the general population, especially from soil and water contamination [50,51].

Overall, the cumulative nature of exposure across multiple routes makes complete avoidance of EDCs difficult, particularly during pregnancy. This underscores the importance of understanding how these compounds interact with biological systems and their potential implications for maternal and fetal health.

### 3.4. Placental Transfer and Fetal Exposure

The placenta is a vital biological connection between the mother and the fetus. It controls the exchange of nutrients and hormones as well as immunological responses. While it acts as a partial barrier to some environmental toxins, endocrine disruptors have been found to cross the placenta and reach the fetus [52,53].

Endocrine disruptors have been detected in the placenta, cord blood, and amniotic fluid, indicating that fetal exposure occurs at early and critical stages of development [54]. Placental transfer is particularly pronounced for lipophilic compounds, including polychlorinated biphenyls (PCBs), dioxins, and certain pesticides, which accumulate in maternal adipose tissue and may be mobilized during pregnancy. Similarly, persistent chemicals such as PFAS have been found to cross the placenta and accumulate in the fetal circulation [55].

Exposure of the fetus to these chemicals is of particular concern, as the developing immune, nervous, and endocrine systems are highly sensitive to external disruptions. Disruption of hormone-related signals during critical “windows” of development can result in permanent physiological alterations, as well as increased risk of disease [56].

Moreover, perinatal exposure to endocrine-disrupting chemicals may directly influence the placenta, causing OS, inflammation, and altered gene expression. These processes may affect maternal–fetal immunological interactions and susceptibility to infection during pregnancy [23,57]. Thus, the mechanisms of placental transfer of endocrine-disrupting chemicals, as well as their effects on fetal development, are of particular interest, as they inform the assessment of exposure risk.

## 4. Immunological Changes During Pregnancy

The immune system of the mother changes in complex and dynamic ways during pregnancy, and this change is characterized by a balance between the need to protect the body from infection and the need to accept the fetus, which is not genetically identical to the mother [58,59]. Nowadays, pregnancy is no longer considered a state of immunosuppression, but rather a complex immunological state in which innate and adaptive immunity are modulated to support fetal development and maternal health [60].

In the initial stages of pregnancy, a regulated inflammatory state is necessary for the success of implantation and placental development, which is essential for trophoblast cell invasion. During this period, innate immune cells, including macrophages, dendritic cells, and natural killer (NK) cells, exhibit increased activity, particularly within the placenta. NK cells in the placenta help regulate blood vessel formation and support fetal development by releasing cytokines and growth factors [61,62,63].

As pregnancy advances, the immunological microenvironment gradually changes to a more anti-inflammatory state, which is favorable for immune tolerance to the fetus and prevents overactivation of the immune response to fetal antigens. The balance between the different types of helper T lymphocytes is a critical component of this immunological adaptation [64]. Pregnancy has traditionally been viewed as a shift from Th1-type immune responses, associated with cellular immunity and inflammation, to Th2-type responses, which promote humoral immunity and anti-inflammatory processes. Although this shift is favorable for the fetus, it may influence the susceptibility of the mother to some infectious agents [65].

Regulatory T cells (Tregs) play a critical role in immune tolerance by suppressing excessive immune activation and promoting tolerance to fetal antigens through the production of anti-inflammatory cytokines, including IL-10 and TGF-β. The normal state of pregnancy is associated with an increase in Tregs, and abnormalities in Treg function have been associated with pregnancy complications, including preeclampsia and recurrent spontaneous abortions [66].

Additionally, changes occur in the innate immune response. During pregnancy, the number of neutrophils and their activity tend to increase, enhancing antimicrobial responses. Monocytes and macrophages also exhibit altered phagocytic activity, while dendritic cells play a crucial role in antigen presentation and in regulating the immune balance between the mother and the fetus [59,67].

These changes are mediated mainly by hormonal influences. Estrogens, progesterone, and hCG have major immunomodulatory effects. Estrogens play a major role in the development and function of immune cells and regulate the production of cytokines. Progesterone promotes the anti-inflammatory response and the development of regulatory immune cells. These hormones regulate the immune response to provide both tolerance and immunity [68,69].

Despite these changes, pregnancy may make the body more vulnerable to certain infections. Viruses like influenza and cytomegalovirus, as well as some bacteria, can lead to more serious pregnancy outcomes due to changes in the immune system [17]. Moreover, environmental factors such as endocrine disruptors may influence the maintenance of immune balance. These EDCs interact with hormone receptors, thereby affecting immune function. Thus, disruption of the immune and endocrine systems due to environmental toxins might compromise the immune system of the pregnant individual [22,23].

Understanding these immunological adaptations is essential, as it helps clarify the role of environmental factors, such as endocrine disruptors, in susceptibility to bacterial and viral infections during pregnancy and contributes to a better understanding of the links between endocrine disruption, the microbiome, and infection risk.

## 5. Impact of Endocrine Disruptors on the Maternal Microbiome

The human microbiome plays a crucial role in regulating immune responses and maintaining normal homeostasis [70]. Microbial communities that reside in various parts of the body, such as the gastrointestinal and reproductive systems, contribute to nutrient metabolism, protection against pathogens, and immune modulation. During pregnancy, the maternal microbiome undergoes dynamic changes that support fetal development and maternal metabolic adaptations [71,72].

Increasing evidence suggests that environmental factors, such as exposure to EDCs, can influence the composition and function of these microbial communities. These substances can directly affect the microbiota by altering microbial growth and metabolism, as well as indirectly through host-related mechanisms, including immune modulation, hormonal changes, and disruption of epithelial barrier integrity [73,74].

Dysbiosis refers to an imbalance in the intestinal microflora, which may compromise host defenses and leave the body vulnerable to infections [75]. In pregnancy, dysbiosis has been linked with enhanced vulnerability to bacterial and viral infections in both the mother and fetus. Exploring the mechanisms by which EDC exposure may be implicated in infectious diseases of pregnancy requires understanding the interactions between environmental chemicals and the maternal microbiome [76].

### 5.1. Microbiome in Pregnancy

Alterations in the maternal microbiome correspond to the physiological and metabolic adaptations that occur during pregnancy. The gut and vaginal microbiomes have attracted considerable research interest due to their roles in immune regulation, metabolism, and pathogen defense [77,78].

The gut microbiome undergoes progressive changes during pregnancy, particularly in the third trimester, characterized by decreased microbial diversity and shifts in the relative abundance of specific bacterial groups, such as Actinobacteria and Proteobacteria. These changes contribute to metabolic adaptations necessary for pregnancy, enhancing energy storage and regulating inflammatory processes [79,80]. Concurrently, the intestinal microbiota influences systemic immune signaling and immune system development through the production of microbial metabolites, including short-chain fatty acids [81].

Similarly, the vaginal microbiota is crucial for maintaining reproductive health. It is normally dominated by *Lactobacillus* species, including *Lactobacillus crispatus*, *Lactobacillus jensenii*, and *Lactobacillus gasseri*, which produce lactic acid, hydrogen peroxide, and antimicrobial peptides to maintain low pH and prevent pathogenic overgrowth [82,83]. Although some studies have detected microbial DNA in placental tissue, the existence of a distinct placental microbiota remains uncertain. However, the gut and vaginal microbiomes influence the maternal–fetal interface [84]. Overall, maternal microbiomes are vital for immune balance and protection against infections. Disruptions in these communities may substantially increase susceptibility to infection, affecting both maternal and fetal health.

### 5.2. EDC-Induced Dysbiosis

Evidence indicates that exposure to endocrine disruptors can significantly affect microbiome composition and function. Experimental and epidemiological studies suggest that phthalates, bisphenols, and persistent organic pollutants can influence microbial diversity and functionality [85].

A major mechanism involves direct interactions with microorganisms, altering growth, metabolism, and bacterial proportions. For example, BPA exposure can specifically alter gut bacteria involved in inflammation and metabolism [86,87].

EDCs can also indirectly influence the microbiome by affecting the host, such as altering hormone levels, immune responses, and secretions essential for maintaining microbial communities [74,88]. Moreover, endocrine disruptors are associated with increased inflammatory responses, altered immune cell activity, activation of OS pathways, and changes in cytokine production. These alterations disrupt host–microbe balance and favor opportunistic pathogens [89].

These effects are particularly relevant in pregnancy, influencing both gut and vaginal microbiota. Altered vaginal microbiota may increase the risk of bacterial vaginosis and other reproductive tract infections, while gut microbiota changes may affect systemic inflammation and immune regulation, impacting the body’s response to infectious agents [90,91]. Collectively, these findings highlight the role of endocrine disruptors as mediators linking microbiota, immune responses, and infection susceptibility during pregnancy. Table 2 summarizes the main classes of EDCs, their direct and indirect effects on the maternal microbiome, and pregnancy-relevant outcomes.

### 5.3. Consequences of Dysbiosis

Microbial dysbiosis during pregnancy has been associated with adverse outcomes for both mother and newborn. Altered microbial community composition can impair colonization resistance, increasing susceptibility to infection and promoting growth of opportunistic pathogens [98,99].

A representative example is bacterial vaginosis, characterized by overgrowth of anaerobic bacteria, including *Gardnerella*, *Prevotella*, and *Atopobium*, and a reduction in protective *Lactobacillus* species. This disorder is linked to pregnancy complications, such as premature rupture of fetal membranes, intrauterine infections, and preterm labor. Environmental factors that disrupt microbial balance may exacerbate these conditions [100,101].

Alterations in gut microbiota may modulate immune function and systemic inflammatory responses. Dysbiosis can decrease production of microbial metabolites essential for controlling host immune responses, impairing the ability to fight bacterial and viral infections. Increased intestinal permeability and inflammation may further compromise immunity [102].

Microbial imbalances at mucosal surfaces, including gut, vagina, and urinary tract, facilitate pathogen colonization and transmission, increasing infection risk. This is especially significant during pregnancy, as the immune system is already adapted to support the fetus [103,104]. Understanding how endocrine disruptors affect microbial communities helps clarify the complex relationships among environmental exposures, immune responses, and infections during pregnancy.

## 6. Endocrine Disruptor Exposure and Bacterial Complications in Pregnancy

Bacterial infections are among the most common complications during pregnancy and can significantly impact maternal health and fetal development. Physiological changes during pregnancy, including hormonal, immunological, and anatomical changes in the urinary system, increase susceptibility to bacterial infections [105,106].

Recent evidence suggests that exposure to EDCs may contribute to the development or worsening of bacterial infections. EDCs can disrupt multiple biological systems, including endocrine signaling, immune regulation, and the microbiome, thereby compromising host defenses and facilitating microbial colonization. Environmental chemical exposures have been linked to increased risk of endometrial infections, bacterial vaginosis, and urinary tract infections [107,108].

These effects arise from interacting mechanisms, including microbial imbalance, immune dysfunction, and epithelial barrier disruption. Understanding the role of endocrine disruptors in bacterial infection susceptibility during pregnancy is particularly relevant for prevention strategies [109].

### 6.1. Urinary Tract Infections (UTIs)

UTIs are among the most common bacterial infections during pregnancy. Pregnant women are at increased risk due to anatomical and physiological changes, including ureteral dilation, decreased bladder muscle tone, and urine retention, partly driven by elevated progesterone. UTIs may present as asymptomatic bacteriuria, cystitis, or, in more severe cases, pyelonephritis [105].

The primary causative agent is *Escherichia coli*, which ascends from the gastrointestinal tract. Other bacteria, including *Enterococcus*, *Proteus mirabilis*, and *Klebsiella pneumoniae*, may also contribute [110]. EDCs may influence UTI susceptibility by altering immune regulation and cytokine signaling, reducing control over bacterial growth. They may also affect gut microbiota composition, increasing potential uropathogens and facilitating colonization of the urinary tract. Alterations in mucosal immunity and epithelial function may further enhance bacterial adhesion and tissue penetration [111].

Experimental studies indicate that environmental chemicals can influence microbial colonization and immune responses relevant to UTIs. However, data on EDCs and UTIs during pregnancy remain limited, and further research is needed [107].

### 6.2. Bacterial Vaginosis and Vaginal Dysbiosis

Bacterial vaginosis (BV) is a common disorder of the vaginal microbiota, characterized by microbial imbalance, overgrowth of anaerobic bacteria, and reduced *Lactobacillus* species. Common causative organisms include *Gardnerella vaginalis*, *Atopobium vaginae*, *Prevotella* species, and *Mobiluncus* species. BV raises vaginal pH and increases susceptibility to infections [112].

In pregnant women, BV is associated with preterm labor, premature rupture of membranes, chorioamnionitis, and post-delivery infections. Maintaining vaginal microbiota stability and adequate *Lactobacillus* levels is essential for reproductive health [100,113].

Evidence suggests that environmental factors, such as EDC exposure, can alter vaginal microbial composition. Chemicals such as phthalates and bisphenols are linked to changes in bacterial diversity and abundance, potentially via hormonal imbalance, immune modulation, and mucosal environment alterations [88,107].

Regulation of the vaginal microbiome is largely estrogen-dependent. Estrogen promotes glycogen accumulation in vaginal epithelial cells, supporting *Lactobacillus* proliferation, lactic acid production, and low pH maintenance. Disruption of this hormonal control by EDCs can promote anaerobic bacterial growth and dysbiosis. EDCs may also alter mucosal defense responses, including cytokine production, antimicrobial peptide expression, and barrier function, increasing infection risk [114,115,116].

### 6.3. Intrauterine and Placental Infections

Placental and fetal membrane inflammation is closely related to intrauterine infections, which are significant pregnancy complications. Pathogens can ascend from the lower genital tract, causing chorioamnionitis and intra-amniotic infection. Common organisms include group B *Streptococcus*, *Ureaplasma*, *Mycoplasma* species, and anaerobic bacteria [117,118].

Inflammatory responses from intrauterine infections may lead to premature rupture of membranes, preterm birth, fetal damage, or neonatal infection. Normally, these infections are limited by the placental barrier and maternal immunity, but microbial and immune imbalances can increase susceptibility [119,120].

EDC exposure may elevate risk by promoting proliferation of pathogenic vaginal organisms, disrupting immune responses, and inducing inflammation. Certain endocrine disruptors have been shown to cause OS and inflammation in the placenta, potentially impairing utero–fetal interface integrity and increasing susceptibility to infection [107,121].

In conclusion, evidence suggests that endocrine disruptors may increase intrauterine infection risk by disrupting microbiome balance, immune regulation, and placental function. Further research is needed to clarify mechanisms and the relationship between environmental exposures and pregnancy complications.

## 7. Endocrine Disruptors and Viral Infections in Pregnancy

Viral infections during pregnancy pose significant risks to both mother and child, as some viruses can cause serious maternal illness or infect the fetus, leading to adverse pregnancy outcomes. Physiological and immunological changes during pregnancy, although necessary to ensure tolerance to the fetus, may impair the body’s ability to effectively counteract viral infections [122,123].

Environmental factors, including EDCs, have been shown to influence antiviral immunity by interacting with hormone receptors, thereby affecting immune and hormonal function. EDCs may also alter host–virus interactions through microbiome disruption and immune modulation [124,125].

Elucidating how endocrine disruptors influence antiviral immunity requires thorough investigation, which can enhance understanding of both EDC-associated risks and pregnant women’s susceptibility to viral infections and related complications [22].

### 7.1. Viral Infections Relevant to Pregnancy

During pregnancy, certain viral infections pose significant risks via maternal infection or vertical transmission to the fetus, potentially leading to congenital abnormalities, intrauterine death, or developmental problems [126,127].

Cytomegalovirus (CMV), a herpesvirus, is among the most common congenital infections worldwide, causing hearing loss and neurodevelopmental issues. Vertical transmission may occur from primary infection or reactivation of latent infection in the mother [128,129].

Influenza virus represents a substantial threat due to physiological changes in respiratory, cardiovascular, and immunological systems, increasing susceptibility to hospitalization, respiratory difficulties, and pregnancy complications [130].

Zika virus, transmitted primarily via mosquitoes, is associated with congenital Zika syndrome, including microcephaly and other neurological disorders. SARS-CoV-2 infection has also raised concern; although most pregnant women experience mild to moderate disease, severe cases can cause placental inflammation and preterm birth. Other viruses of concern include hepatitis, rubella, and varicella-zoster [131,132].

The severity of viral infections varies with pregnancy stage and immune response efficiency. Therefore, it is crucial to identify factors, including environmental exposures, that influence susceptibility.

### 7.2. Immunomodulatory Effects of EDCs

EDCs can influence antiviral immunity via hormonal signaling pathways. Disruption of hormones such as progesterone, estrogen, and thyroid hormones may compromise antiviral defense [22,125].

Certain EDCs affect interferon production, a critical component of antiviral immunity, impairing the body’s ability to clear infections. Key innate immune cells, including NK cells, macrophages, and dendritic cells, may also be compromised, leading to delayed viral clearance and increased infection susceptibility [133].

EDCs have been linked to OS and chronic low-grade inflammation, which can further impair immune regulation and create conditions favorable for viral persistence or increased disease severity. Interactions with the microbiome also play a role, as microbiota modulate immune signaling and response [134]. In summary, heightened vulnerability to viral infections during pregnancy is mediated through the interplay of endocrine disruption, immune dysregulation, and microbiome alterations.

### 7.3. Evidence Linking EDC Exposure to Viral Susceptibility

Evidence links EDC exposure to alterations in antiviral immunity, though research is still in early stages. Epidemiological and experimental studies suggest that environmental contaminants can alter immune function, critical for preventing viral infections [135,136].

For instance, PFAS exposure is associated with reduced antibody production post-vaccination, suggesting modulation of adaptive immunity. BPA has been shown to affect cytokine production and immune signaling, and animal studies indicate BPA alters expression of immune-related genes. Pesticides and persistent organic pollutants are also linked to immunotoxicity, weakening defenses via altered inflammation, cytokine signaling, and immune cell function [137,138,139].

Despite these findings, epidemiological evidence directly linking EDC exposure to viral infection outcomes in pregnancy remains limited. Current data suggest that environmental chemicals disrupting endocrine and immune function may alter susceptibility to viral infections [135,140].

**Figure 1:**
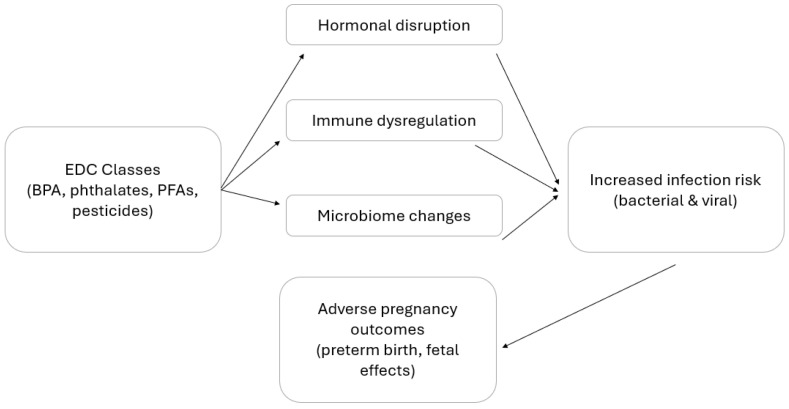
Overview of EDC-related pathways and infection risk in pregnancy. EDCs affect hormonal, immune, and microbiome pathways, increasing susceptibility to bacterial and viral infections during pregnancy and contributing to adverse maternal and fetal outcomes.

Further research is imperative to clarify the complex relationships among endocrine disruption, immune regulation, environmental exposures, and viral infection risk during pregnancy [23]. Table 3 summarizes viral infections relevant to pregnancy and the mechanisms by which EDCs may influence susceptibility. An overview of the relationship between endocrine-disrupting chemicals, key biological pathways, and infection-related outcomes in pregnancy is presented in Figure 1.

## 8. Mechanistic Pathways Linking EDCs to Infection Susceptibility

To understand the effects of EDCs on the health of pregnant women and their babies, it is important to investigate how these chemicals influence susceptibility to infectious diseases [22,29,148].

One main effect of EDCs is disruption of hormonal regulation. During pregnancy, proper hormonal control is critical for immune homeostasis. EDCs can affect hormones such as estrogen, androgen, and thyroid hormones by binding to hormone receptors and altering their activity [26,149].

Immune dysregulation is another important pathway. Both innate and adaptive immunity undergo substantial and dynamic changes throughout pregnancy. Changes in cytokine networks, including elevated pro-inflammatory mediators such as TNF-α and IL-6, as well as alterations in anti-inflammatory pathways (e.g., IL-10), have been linked to exposure to EDCs [150]. These changes may contribute to an imbalance in T-cell subsets, including shifts in Th1/Th2 responses and alterations in regulatory T cells (Treg), which are essential for maintaining immune tolerance during pregnancy.

Furthermore, EDCs may modulate innate immune signaling pathways, including Toll-like receptor (TLR) activation, thereby altering pathogen recognition and the initiation of immune responses. Interactions with endocrine receptors, such as estrogen and androgen receptors, further influence immune cell function and inflammatory signaling. Collectively, these mechanisms can impair pathogen defense and increase susceptibility to infection [10].

EDCs also influence the microbiome. They may act directly on microbial communities by altering growth and metabolism or indirectly by affecting the host, including hormonal and immune status and epithelial integrity. Such changes can reduce colonization resistance and increase susceptibility to infection, particularly in the gut and vaginal microbiomes [74,107,151].

Furthermore, several EDCs induce OS and chronic inflammation. Reactive oxygen species generation and disruption of antioxidant defenses can impair cellular structures and trigger inflammatory responses. Chronic inflammation may compromise tissue barriers and immune responses, further compromising host defense mechanisms [22].

The impact of EDCs on placental function is also significant. The placenta serves as a critical tissue barrier and immunological interface [152]. Abnormalities in placental vasculature, inflammation, and gene expression associated with environmental pollutants may impair immunological interactions and damage placental barrier function, facilitating intrauterine infection process [153].

Genetic variation is another factor influencing vulnerability to EDCs. The metabolism and removal of EDCs may be considerably impacted by variations in genes encoding detoxification enzymes, such as cytochrome P450 (CYP450), glutathione S-transferases (GST), and UDP-glucuronosyltransferases (UGT) [29,52,96]. Immune regulation, OS reactions, and internal chemical load can all be affected by variations in these pathways [29,56]. Therefore, inter-individual variation in susceptibility to infections during pregnancy after EDC exposure may be partially explained by genetic background [29]. Further research in this area is crucial, despite the current lack of data in pregnant populations.

These mechanisms are closely related to one another rather than operating independently. Cytokine production, immune cell activity, and inflammatory responses can all be affected by endocrine disruption induced by exposure to EDCs [10,124,136]. The microbiome’s composition and stability may be impacted by these immunological changes, which may lower colonization resistance and promote the emergence of opportunistic infections. Immune dysregulation may be exacerbated by microbiome disruption, resulting in a feedback loop that increases susceptibility to bacterial and viral infections [89,136]. The intricate interactions between the immunological, microbial, and endocrine systems that determine the risk of infection during pregnancy are highlighted by this integrated viewpoint [22,28,78,104,136].

**Figure 2:**
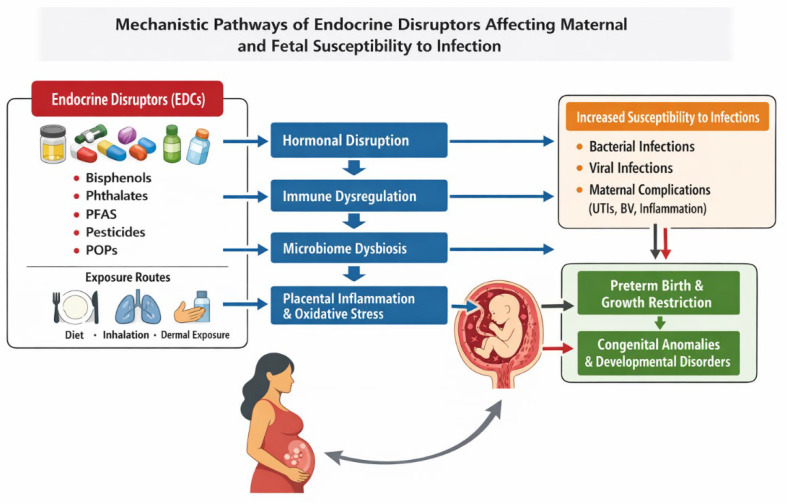
Mechanistic pathways by which EDCs affect maternal and fetal susceptibility to infection. EDCs (bisphenols, phthalates, PFAS, pesticides, POPs) act via multiple maternal targets, including hormonal disruption, immune dysregulation, microbiome dysbiosis, and placental inflammation/OS. These maternal effects increase susceptibility to bacterial and viral infections and contribute to adverse pregnancy outcomes, including preterm birth, growth restriction, congenital anomalies, and developmental disorders. Exposure routes (diet, inhalation, dermal contact) are also depicted.

Collectively, these mechanisms illustrate the complex interplay between endocrine disruption, immune regulation, microbiome balance, inflammation, and placental function. Understanding these pathways is essential for elucidating the role of environmental exposures in infection development during pregnancy and informing effective preventive strategies [154]. Figure 2 illustrates the mechanistic pathways by which EDCs influence maternal immune regulation, microbiome balance, placental function, and fetal susceptibility to infections.

## 9. Implications for Maternal and Fetal Health

Exposure of pregnant women to EDCs can cause serious health consequences for both the mother and the fetus [155]. These include disruption of critical biological processes such as placental function, immune regulation, endocrine activity, and the microbiome, which may increase susceptibility to infections during this sensitive period [104].

Disruptions in the endocrine and immune systems may alter cytokine production, reduce immune function, and modify inflammatory responses. This can increase the risk of bacterial vaginosis, urinary tract infections, and viral infections, potentially leading to systemic inflammation, hospitalization, or sepsis [156].

These effects extend to the fetus. Pregnancy-related infections are associated with premature birth, premature rupture of membranes, and growth restriction, while viral infections may lead to congenital anomalies, developmental disorders, and neurological problems. Environmental factors that increase infection risk thus indirectly affect fetal health [157,158,159].

The placenta plays a central role in maternal–fetal communication. EDC exposure is associated with inflammation, OS, and altered placental gene expression, potentially compromising barrier integrity and facilitating pathogen invasion [53].

Evidence also suggests that early-life exposure to environmental pollutants and inflammatory reactions may have long-term effects on children’s health. According to the “developmental origins of health and disease” (DOHaD) concept, this may include impacts on the immune system, metabolism, and the risk of chronic diseases [23].

In conclusion, understanding how endocrine disruptors influence susceptibility to infections during pregnancy is critical for protecting maternal and fetal health. Raising awareness of EDC risks and conducting further research on the interactions among environmental factors, immune regulation, the microbiome, and infections will help inform effective preventive strategies.

## 10. Research Gaps and Future Perspectives

Despite evidence suggesting that EDCs may affect the immune system, microbiome, and infection risk, significant knowledge gaps remain. The relationship between EDC exposure and infection risk during pregnancy is complex and not fully understood. Addressing these gaps is essential to improve risk assessment and develop strategies that safeguard maternal and fetal health [29].

Although the available literature provides valuable insights, it is important to consider variability in study design and evidence quality. Although human epidemiological studies provide clinically relevant information, they are often constrained by confounding variables, inconsistent exposure assessment, and challenges in establishing causal relationships [44,50,124]. Animal and in vitro research, on the other hand, offers valuable mechanistic insights but might not accurately mimic human physiological conditions [29,33]. Variability between research is further influenced by variations in sample size, exposure timing, and outcome definitions; in certain instances, contradictory results have been reported [25,28,47]. These limitations highlight the importance of well-designed longitudinal human studies and the need for cautious interpretation of findings [29,50].

A major limitation in current literature is the lack of longitudinal studies on human populations examining the link between EDC exposure and infection risk. Most studies investigate discrete aspects of this association and fail to integrate data into a unified framework for comprehensive understanding [9].

Real-world exposure is complex, as humans encounter mixtures of chemicals rather than single compounds. However, most toxicological studies focus on individual substances. Future research should examine the combined and potentially synergistic effects of multiple EDCs to more realistically reflect human exposure [47].

The quantitative evaluation of EDC exposure is another crucial factor. To determine internal exposure levels, several studies use biomarkers found in biological samples such as blood, urine, or placental tissue [50,52,121]. Comparisons between studies are made more difficult, nevertheless, by differences in exposure measurement techniques and timing [29,50,124]. Moreover, endocrine disruptors frequently exhibit non-monotonic dose–response relationships, whereby low-dose exposures may exert significant biological effects that are not predicted by conventional toxicological models [45,149]. Determining exposure limits and connecting observed concentrations to clinically significant outcomes, such as infection susceptibility and unfavorable pregnancy events, is made more difficult by these features [45,124]. To better understand dose–response interactions in pregnant populations, exposure measurement and longitudinal monitoring must be standardized [29,50].

Modern multi-omics technologies, such as metagenomics, transcriptomics, proteomics, and metabolomics, offer opportunities to better understand the mechanisms by which environmental chemicals affect the immune system, microbiome, and metabolism. Coupling these technologies with epidemiological studies can aid in identifying reliable biomarkers of exposure and early risk indicators [29].

Further investigation of the relationship between EDC exposure and the maternal microbiome is required. The exact mechanisms by which environmental toxins impact microbial diversity remain unclear. Understanding how dysbiosis influences pathogen colonization and host–microbe interactions can provide insights into infection susceptibility. Similarly, the effects of EDCs on placental function and immunological control at the utero–fetal interface remain critical areas of research [23].

Finally, emphasis should be placed on establishing public health policies and preventive measures to reduce exposure to endocrine disruptors. Promoting safer products, increasing public awareness, and restricting hazardous substances can collectively help mitigate risk [160].

Collectively, elucidating these complex interactions provides a foundation for developing interventions that protect maternal and fetal health in an environment burdened by chemical exposures.

## 11. Conclusions

Environmental pollutants known as EDCs can disrupt immune system control, hormone function, and the body’s microbial balance, predisposing the mother to various infections and potentially worsening pregnancy outcomes.

Current evidence indicates that EDC exposure may alter maternal microbiota, cytokine production, and immune regulation, increasing susceptibility to immunosuppression, microbial imbalance, and bacterial and viral infections.

The precise mechanisms by which endocrine disruptors mediate this heightened susceptibility remain unclear. Understanding the complex interplay among the immune system, microbiome, and endocrine system requires further research to develop effective preventive strategies that protect maternal and fetal health.

Nonetheless, it is important to recognize the limitations of the current evidence. Establishing causal relationships is challenging, as much of the available data derives from heterogeneous study designs, including observational studies and experimental models. The interpretation of results is further complicated by difficulties in precisely determining exposure levels and the consequences of combined chemical exposures. Future research should prioritize well-designed longitudinal human studies to better elucidate the relationships between EDC exposure, immune function, microbiome dynamics, and infection risk during pregnancy.

## Figures and Tables

**Table 1:** Major classes of EDCs, their common sources, primary biological effects, and pregnancy-relevant considerations.

EDC Class	Examples	Common Sources	Key Biological Effects	Pregnancy-Relevant Notes
Bisphenols [30,37]	BPA [30,37]	Plastics, epoxy resins, food containers	Estrogenic activity, endocrine disruption	Detected in maternal blood, urine, amniotic fluid, placenta
Phthalates [38,39]	DEHP, DBP [38,39]	Plastics, cosmetics, personal care products	Anti-androgenic, immune modulation	Affect reproductive and immune system development
PFAS [40,41]	PFOA, PFOS [40,41]	Non-stick cookware, stain-resistant fabrics, firefighting foams	Persistent, immune suppression, metabolic disorders	Cross placenta, accumulate in fetal circulation
Pesticides [42,43]	Organophosphates, carbamates, organochlorines [42,43]	Agriculture, soil, water	Thyroid, estrogenic, androgenic disruption	Can leach into food/water, cross placenta
POPs [44,45]	PCBs, dioxins [44,45]	Industrial byproducts, waste incineration	Lipophilic, immune and endocrine disruption	Accumulate in adipose tissue, mobilized during pregnancy

**Table 2:** Effects of EDCs on the maternal microbiome and pregnancy outcomes.

EDC Class	Direct Effects on Microbiota	Indirect Effects (Host-Mediated)	Pregnancy-Relevant Outcomes
Bisphenols [86,92]	Alters gut bacterial composition, including ↑ inflammation-associated taxa (e.g., Proteobacteria) [86,92]	Alters hormone levels, ↑ IL-6, TNF-α, epithelial barrier disruption	Increased gut dysbiosis, systemic inflammation, risk of bacterial and viral infections
Phthalates [93,94]	Changes microbial diversity including ↓ *Lactobacillus* spp. [93,94]	Immune modulation, hormone alterations, cytokine imbalance	Dysbiosis in gut and reproductive tract, increased susceptibility to BV, UTIs
POPs [95,96]	Alters microbial growth and metabolism, reduced beneficial commensals [95,96]	OS, chronic inflammation, immune activation pathways	Reduced colonization resistance, increased pathogen proliferation, higher infection risk
Combined EDC exposure [97,98]	Disruption of microbial community balance [97,98]	Altered cytokine production (IL-6, TNF-α), immune dysregulation	Gut and vaginal dysbiosis, potential adverse pregnancy outcomes including preterm labor, intrauterine infections

**Table 3:** Viral infections relevant to pregnancy and the effects of EDCs on susceptibility.

Virus	Maternal Risks	Fetal/Neonatal Risks	EDC-Mediated Mechanisms	Representative EDCs
Cytomegalovirus (CMV) [22,141]	Hearing loss, neurodevelopmental issues (maternal infection)	Vertical transmission, congenital infection, neurodevelopmental problems	Altered interferon signaling, ↓ antiviral response, immune cell dysregulation	BPA, PFAS, POPs
Influenza [142]	Respiratory complications, hospitalization	Preterm birth, pregnancy complications	Hormone disruption affecting antiviral defense, impaired cytokine response (IL-6, TNF-α)	PFAS, phthalates
Zika virus [143]	Minimal maternal illness in most cases	Congenital Zika syndrome, microcephaly, neurological disorders	Immune dysregulation, altered cytokine signaling, microbiome disruption	BPA, pesticides
SARS-CoV-2 [144,145]	Severe disease in some cases, respiratory complications	Placental inflammation, preterm birth	OS, chronic inflammation, ↑ pro-inflammatory cytokines (IL-6, TNF-α), immune suppression	PFAS, BPA, POPs
Hepatitis, Rubella, Varicella-Zoster [146,147]	Maternal illness	Congenital anomalies, developmental disorders	Hormonal and immune modulation, altered antiviral immune responses	Various EDCs (PFAS, pesticides)

## Data Availability

No new data were created or analyzed in this study.

## Abbreviations

The following abbreviations are used in this manuscript:

EDCs	Endocrine-Disrupting Chemicals
OS	Oxidative Stress
BPA	Bisphenol A
PFAS	Per- and Polyfluoroalkyl Substances
POPs	Persistent Organic Pollutants
OCPs	Organochlorine Pesticides
UTIs	Urinary Tract Infections
BV	Bacterial Vaginosis
CMV	Cytomegalovirus
NK cells	Natural Killer Cells
Tregs	Regulatory T Cells
hCG	Human Chorionic Gonadotropin
DOHaD	Developmental Origins of Health and Disease
PCBs	Polychlorinated Biphenyls
SARS-CoV-2	Severe Acute Respiratory Syndrome Coronavirus 2
Th1	T Helper 1 Cells
Th2	T Helper 2 Cells
PFOS	Perfluorooctanesulfonic Acid
PFOA	Perfluorooctanoic Acid
